# VACTERL/VATER Association

**DOI:** 10.1186/1750-1172-6-56

**Published:** 2011-08-16

**Authors:** Benjamin D Solomon

**Affiliations:** 1 Medical Genetics Branch, National Human Genome Research Institute, National Institutes of Health, Building 35/Room 1B-207, Bethesda, MD 20892, USA

## Abstract

VACTERL/VATER association is typically defined by the presence of at least three of the following congenital malformations: vertebral defects, anal atresia, cardiac defects, tracheo-esophageal fistula, renal anomalies, and limb abnormalities. In addition to these core component features, patients may also have other congenital anomalies. Although diagnostic criteria vary, the incidence is estimated at approximately 1 in 10,000 to 1 in 40,000 live-born infants. The condition is ascertained clinically by the presence of the above-mentioned malformations; importantly, there should be no clinical or laboratory-based evidence for the presence of one of the many similar conditions, as the differential diagnosis is relatively large. This differential diagnosis includes (but is not limited to) Baller-Gerold syndrome, CHARGE syndrome, Currarino syndrome, deletion 22q11.2 syndrome, Fanconi anemia, Feingold syndrome, Fryns syndrome, MURCS association, oculo-auriculo-vertebral syndrome, Opitz G/BBB syndrome, Pallister-Hall syndrome, Townes-Brocks syndrome, and VACTERL with hydrocephalus. Though there are hints regarding causation, the aetiology has been identified only in a small fraction of patients to date, likely due to factors such as a high degree of clinical and causal heterogeneity, the largely sporadic nature of the disorder, and the presence of many similar conditions. New genetic research methods offer promise that the causes of VACTERL association will be better defined in the relatively near future. Antenatal diagnosis can be challenging, as certain component features can be difficult to ascertain prior to birth. The management of patients with VACTERL/VATER association typically centers around surgical correction of the specific congenital anomalies (typically anal atresia, certain types of cardiac malformations, and/or tracheo-esophageal fistula) in the immediate postnatal period, followed by long-term medical management of sequelae of the congenital malformations. If optimal surgical correction is achievable, the prognosis can be relatively positive, though some patients will continue to be affected by their congenital malformations throughout life. Importantly, patients with VACTERL association do not tend to have neurocognitive impairment.

## Disease name and synonyms

VACTERL association (ORPHA887)

VATER association (ORPHA887)

## Definition and diagnostic criteria

VATER association was first named in the early 1970's. As initially described, the condition included the statistically non-random co-occurrence of a group of congenital malformations: Vertebral defects, Anal atresia, Tracheo-Esophageal fistula (TEF) with esophageal atresia, and Radial and Renal dysplasia [1,2]. Because these malformations were observed to occur together more often than would be expected by chance, the condition was termed an association. However, there was not (and still remains no) evidence for a single, unifying cause that would result in the condition being termed a syndrome. One explanation for the clustering of features involves the idea of the "developmental field defect", in which malformations that occur in blastogenesis tend to result in polytopic anomalies, or birth defects affecting multiple organ systems. Some authors have suggested that VACTERL association would be more accurately described as a "primary polytopic developmental field defect" (as this reflects the causative developmental perturbation) rather than an association (as this simply describes the presence of statistical clustering) [3,4].

Shortly after the initial description, it was proposed that the diagnostic criteria should also include Vascular anomalies (as part of the "V" in VACTERL), including single umbilical artery, or SUA, as part of the definition. Cardiac malformations ("C") and additional Limb ("L") anomalies other than strict radial anomalies were added as well [5,6]. Later, statistical analyses of cohorts of affected patients suggested that there was overall less evidence for the inclusion of certain features, such as cardiac or renal anomalies [7-9]. There is still no firm consensus regarding strict diagnostic criteria, though most clinicians and researchers require the presence of at least three component features for diagnosis (although it may be a semantic point, some would argue that the condition is observed rather than truly diagnosed, and while this may be an important distinction, the term "diagnosis" and its derivatives will be used throughout this article). Others have stressed emphasis on certain "core" features such as TEF or anorectal malformations (ARM) [10]. Equally importantly, as the differential diagnosis is quite broad, careful clinical and laboratory-based analysis should not yield evidence of an alternative diagnosis (see the Differential diagnosis section below). For further details regarding each component feature, see the Clinical description section below.

## Epidemiology

As various studies have used differing diagnostic criteria and ascertainment methods, the incidence is difficult to pinpoint. Studies have estimated the frequency to be between less than 1/10,000 to 1/40,000 infants (approximately <1-9/100,000 infants) [8,11,12]. However, care must be taken when interpreting these values. First, different studies have used differing diagnostic criteria. Second, some larger studies extracted data from large malformation registries. These studies, while extremely valuable, may not be designed to be able to strictly rule-out patients with similar but distinct conditions. Along these lines, many older studies were not able to test for similar conditions for which genetic testing is now available, such as Fanconi anemia or chromosomal anomalies detectable on karyotype or microarray. Both of these types of studies would likely overestimate the incidence of VACTERL association, which is likely the reason that some estimates make the condition appear much more common than it likely is in reality. On the other hand, studies based on patients who survive to present to clinics or participate in research studies underestimate the incidence, as only less severely-affected patients are included. Finally, it must be noted that due to modern surgical techniques and specialized hospitalization units (such as neonatal intensive care units), infants born with VACTERL association today have a much better prognosis than several decades ago, which would skew epidemiological data in many types of studies, especially if only infants that survive the immediate postnatal period are included.

There is overall no strong evidence for an increased incidence of VACTERL association in certain areas of the world or in specific ethnic populations. Relatively large cohorts of patients have been described from all parts of the world in which such studies have been conducted [7-17]. Some but not all studies have suggested that the condition is more common in males. As the causes of VACTERL association appear to be heterogeneous, explanations of male overrepresentation in certain cohorts (in addition to chance) may include X-linked inheritance in some instances, sex-influenced expression, and mechanisms related to imprinting defects [11,12,15,16].

As VACTERL association is likely highly heterogeneous, grouping patients through phenotypic and statistical analyses is a critical step in both defining the overall range of manifestations as well as for the exploration of potential causes. Such grouping relies heavily upon careful and thorough clinical examination, and is important for more than descriptive purposes: clustering phenotypically similar patients can enhance consideration of the differential diagnosis and may reveal a group of patients with features that overlap but are distinct from VACTERL association [15-17]. Such analyses can also suggest common, biologically-linked causes within each cluster, as aberrations affecting developmentally linked processes may hypothetically result in similar clinical features [7,15-17]. Determining the type and range of findings may also provide insight into the specific type and temporospatial nature of the underlying biological perturbation, which can inform basic and translational studies regarding the pathogenic mechanism. Additionally, using cluster analysis to inform studies into causation greatly enhances the potential for success through newer gene discovery techniques such as whole-exome sequencing [18]. Finally, clustering of phenotypically similar patients can allow better prognostic discussions and medical management decisions (such as highlighting important clinical features that may be more common in patients within a certain cluster) even when the cause is unknown.

Several studies have shown evidence for clinically-defined clusters depending on the type and spatial location (eg, "upper vs. lower") of VACTERL association component features, as well as the presence of associated congenital anomalies [7,15-17]. However, as described below (in the section on Diagnosis and diagnostic methods), these clusters may also reflect variable diagnostic criteria, and accurate assignment of individual patients to a given cluster may be complicated by issues such as variable expressivity. Once the causes of the condition are better delineated, categorization of patients may be more easily accomplished, and re-analyzing previously purported clusters will be an interesting endeavor [7,15-17].

## Clinical description

VACTERL association is clinically defined by the presence of a cluster of congenital malformations. As described above, most (but not all) clinicians and researchers require the presence of at least three component features, though some place more emphasis on certain component features [7-9,11-15,19]. Importantly, there must be no clinical or laboratory-based evidence of an alternate diagnosis. The following is a discussion of the specific defining features of VACTERL association as the condition is most commonly described. However, it is important to note that the literature demonstrates a wide degree of interpretation in terms of how diagnostic criteria are defined and applied, and any patient with suspected VACTERL association deserves careful scrutiny.

In several studies, vertebral anomalies, which are commonly accompanied by rib anomalies, have been reported in approximately 60-80% of patients; interestingly, patients may have rib anomalies without vertebral anomalies [7-9,13,15,20]. Vertebral anomalies typically include segmentation defects, such as hemivertebrae, "butterfly vertebrae", "wedge vertebrae" (the latter two descriptions refer to the shape of the dysplastic vertebrae), and vertebral fusions, supernumerary or absent vertebrae, and other forms of vertebral dysplasia. There is a wide degree of severity of reported vertebral malformations. Some patients require multiple, major operations for vertebral anomalies, while others may have subtle defects only detectable through careful scrutiny by an experienced clinician [13,21]. Abnormal spinal curvature due to underlying costovertebral anomalies is common. Clinical signs of scoliosis may be the first sign of vertebral anomalies if imaging studies are not performed when VACTERL association is first suspected [22]. Of note, while patients with ARM may have dysplastic sacral vertebrae, whether or not these should be included as true vertebral malformations for the diagnosis of VACTERL association is unclear [13,21,23]. In fact, while these latter patients are sometimes described as having VACTERL association (especially if a minor renal anomaly is present), such a diagnosis is controversial.

Imperforate anus/anal atresia as part of an ARM occurs in approximately 55-90% of patients [7-9,13,15]. A complete imperforate anus is often discovered in the immediate postnatal period, typically through routine examination or due to inability to measure the infant's temperature rectally. However, other forms with stenosis may appear anatomically normal on initial examination, and may clinically present later with signs of obstruction [23]. (While beyond the scope of this paper, formal classification schemes of many of the malformations seen in VACTERL association, such as ARM and TEF are available; please see specific references for more details.) As with other malformations, some controversy exists in terms of diagnostic criteria. For example, some clinicians only allow a completely imperforate anus to be considered part of VACTERL association (though this author would argue that this is too narrow a view, and that some allowance should be made for a spectrum of severity).

In patients with imperforate anus, genitourinary (GU) anomalies are also common as part of the ARM, but GU anomalies may also occur in patients without imperforate anus or anal atresia. Overall, GU anomalies occur in up to 25% of patients with VACTERL association, and may be less obvious than imperforate anus, such as is the case with fistulae connecting the GU and anorectal tracts [23,24].

Cardiac malformations have been reported in approximately 40-80% of patients with VACTERL association [9,13,15]. One explanation for the highly variable rates of cardiac malformations may have to do with ascertainment bias in certain studies. For example, studies using malformation registries tend to describe higher rates of cardiac malformations than studies based on children seen in genetics clinics, as severe cardiac malformations result in a high rate of mortality. It is important to point out that some statistically-based analyses argue that cardiac malformations should not be included in the diagnostic criteria, as they are not more common in patients with VACTERL association than in other disorders with multiple malformations [7-9]. Nevertheless, structural heart anomalies are common in VACTERL association, and may range from severe structural defects incompatible with life or necessitating several stages of challenging surgery, to subtle anatomic defects ascertained in adulthood only through participation in research studies, and which would not be expected to cause any medical issues [13,15,22]. The category of cardiac defects raises another important point that might be applied to VACTERL association more generally: certain variants in isolation (such as patent ductus arteriosus or patent foramen ovale) should standardly be considered a normal, age-based finding rather than a component feature of VACTERL association, and researchers and clinicians must be careful not to apply diagnostic criteria carelessly.

A number of subtypes of tracheo-esophageal fistula (TEF) may occur, and may present with or without esophageal atresia. Overall, TEF occurs in approximately 50-80% of patients [7-9,13,15]. Early signs of TEF include polyhydramnios or absent gastric bubble recognized prenatally, inability to pass nasogastric tubes immediately postnatally, or choking/swallowing in infancy [25]. TEF typically require surgery in the first few days of life, and later complications may occur as well, such as fistula recurrence, reactive-airway disease, and gastro-esophageal reflux [22]. In addition to TEF, other pulmonary anomalies may co-occur; these may share a common structural anatomical cause with the TEF and/or cardiac anomalies [16,26-29].

Like other malformations seen in VACTERL association, there can be a wide range of severity and type of renal anomalies, which can include unilateral renal agenesis (or bilateral in severe cases), horsehoe kidney, and cystic and/or dysplastic kidneys, sometimes accompanied by ureteral and GU anomalies [15,24]. Renal anomalies are reported in approximately 50-80% of patients [8,9,13,15]. As with cardiac malformations, some statistical analyses have suggested that renal anomalies should not be considered one of the defining component features, as they may only be associated with certain features such as ARM [7-9]. Unlike many other features of VACTERL association, which are relatively clinically obvious, renal anomalies may be less apparent unless careful imaging is performed. Diagnosing occult renal anomalies is especially important, as these malformations may result in significant morbidity [30].

Finally, limb malformations have been reported in approximately 40-50% of patients [7-9,15,17]. While classically defined as radial anomalies, including thumb aplasia/hypoplasia, many other limb anomalies have been ascribed (perhaps erroneously) to VACTERL association, including polydactyly and lower limb anomalies. As with the other malformations, there is a wide range of the degree of severity of limb anomalies in affected patients. One must take care to consider whether the particular limb anomaly in question should be considered part of VACTERL association or be taken to be a sign of a similar disorder in the differential diagnosis.

While the above malformations are considered to be the core component features, many other malformations have been described in affected patients [8,9,15,31]. On review, some of these features are likely signs of other, similar disorders, such as the finding of craniosynostosis in patients with Baller-Gerold syndrome, or coloboma in patients with CHARGE syndrome [13,15,19,32,33]. Clinicians should thus use these non-typical malformations as a clue in considering possible other conditions, and should be cautioned to look carefully at other organ systems that could aid in the differential diagnosis, such as by obtaining ophthalmologic and audiologic examinations (see Table 1).

**Table 1:** Differential diagnosis: conditions with multiple features in common with VACTERL association.

Condition	Features in common with VACTERL association	Features distinct from VACTERL association	Cause(s)	Reference(s)
Alagille syndrome	Vertebral anomalies, cardiac anomalies; may have renal anomalies	Bile duct paucity and cholestasis, ophthalmologic anomalies (especially posterior embryotoxon), neurological anomalies, characteristic facial appearance	Heterozygous mutations in *JAG1*, *NOTCH2*	[92-95]

Baller-Gerold syndrome	Radial anomalies, may also include anal anomalies	Craniosynostosis, skin anomalies	Heterozygous mutations in *RECQL4*	[33]

CHARGE syndrome	Cardiac malformations, genitourinary anomalies; may also include TEF	Colobomata, choanal atresia, neurocognitive and growth impairment, ear anomalies, cranial nerve dysfunction, characteristic facial features	Heterozygous mutations in *CHD7*	[32,96]

Currarino syndrome	Sacral malformations, ARM	Presacral mass	Heterozygous mutations/ deletions of *HLXB9*	[97,98]

22q11.2 deletion syndrome (also known by other names, such as DiGeorge syndrome or velocardio-facial syndrome)	Cardiac malformations, renal anomalies, other VACTERL-type anomalies also reported	Hypocalcemia, palatal anomalies, learning difficulties, immune dysfunction, neuropsychiatric disturbances, characteristic facial features,	Deletion of one copy of chromosome 22q11.2	[99]

Fanconia anemia	Virtually all features of VACTERL association may occur; radial anomalies are considered an especially key feature	Hematologic anomalies, pigmentation anomalies	Recessive or X-linked mutations in multiple genes; typically detected by chromosomal breakage studies	[62,63,66,100]

Feingold syndrome	GI atresia, cardiac defects, renal anomalies	Brachymesophalangy, toe syndactyly, microcephaly, cognitive impairment, characteristic facial appearance,	Heterozygous mutations in *MYCN*	[67,101]

Fryns syndrome	GI malformations, cardiac defects, GU anomalies	Diaphragmatic defects, neurocognitive impairment, characteristic facial appearance,	No well-characterized unifying causes	[102]

Holt-Oram syndrome	Cardiac malformations, limb malformations	Cardiac conduction disease (also reported in VACTERL association)	Heterozygous mutations in *TBX5*	[68,103]

Müllerian duct aplasia, renal aplasia, and cervico-thoracic somite dysplasia (MURCS assoc-iation); also known as Mayer-Rokitansky- Küster-Hauser syndrome type II	Vertebral anomalies, renal anomalies, GU anomalies and anorectal malformations; may also have cardiac and limb anomalies	Syndactyly and hearing loss have been described	Unknown; likely heterogeneous	[104,105]

Oculo-auriculo-vertebral syndrome	Vertebral anomalies, cardiac abnormalities, limb abnormalities, urogenital anomalies	Ear anomalies (microtia), hemifacial microsomia, neurocognitive impairment, facial clefts (also described in patients with VACTERL association)	Unknown; likely heterogeneous	[106]

Opitz G/BBB syndrome	Anal anomalies, heart defects, TEF, hypospadias	Hypertelorism, syndactyly	X-linked form: heterozygous/ hemizygous mutations in *MID1*; autosomal dominant form: some cases due to deletion 22q11.2	[107-109]

Pallister-Hall syndrome	Imperforate anus, renal anomalies, limb anomalies (postaxial polydactyly should serve as a clue for the Pallister-Hall syndrome)	Hypothalamic hamartoma, bifid epiglottis (ranging to more severe types of clefts), nail hypoplasia	Heterozygous mutations in *GLI3 *	[110-112]

Townes-Brocks syndrome	Imperforate anus, thumb anomalies, renal anomalies, cardiac anomalies	Dysplastic ears, hearing loss	Heterozygous mutations in *SALL1 *	[69,113]

VACTERL-H	All core component features	Hydrocephalus	Heterozygous mutations in *PTEN*, heterozygous/ hemizygous mutations in *ZIC3*; X-linked and recessive forms have been described	[64,65,90,114]

There are also many scattered case reports in the medical literature describing additional patients who have features observed in VACTERL association, but only the main overlapping conditions are described here.

GI: gastrointestinal; GU: genitourinary; H:hydrocephalus; TEF: tracheo-esophageal fistula

Special note should be made of the presence of a single umbilical artery, which is frequent in patients with VACTERL association (though the exact prevalence is difficult to estimate) [31,34]. This is an especially important antenatal finding, as it may be the first sign of the diagnosis (see the section on Antenatal diagnosis below).

## Aetiology

In at least a subset of patients, there is evidence for familial clustering suggestive of inherited factors [35-37]. However, there is also strong clinical and genetic evidence for causal heterogeneity in patients with VACTERL association [12,15-17]. The vast majority of genetic causes described in humans have been reported in isolated individuals or families and overall account for only a small percentage of patients with VACTERL association. These causes are outlined in Table 2.

**Table 2:** Reported causes of features seen in VACTERL association in human patients.

Cause	Notes	Reference(s)
Mitochondrial dysfunction	Patients typically have clinical features consistent with mitochondrial dysfunction (though these may not be apparent until long after the malformations associated with VACTERL association have been discovered)	[77-81]

Pathogenic copy number variations	Many different deletions/duplications have been reported*, though the evidence for causation of VACTERL association-type features is not uniformly clear. Clinical features in patients with large genomic imbalances often include malformations and medical issues not commonly seen in VACTERL association (such as neurocognitive impairment)	[82-86]

Heterozygous mutations in *HOXD13 *	Described in one patient; mutations in *HOXD13 *are more typically reported as resulting in limb and/or urogenital anomalies	[51,87,88]

Heterozygous/ hemizygous mutations in *ZIC3 *	Clinical features may or may not include obvious heterotaxy/situs abnormalitites	[89-91]

*A number of references have been listed here, but this is not an exhaustive list.

In addition to the small handful of proposed human etiologies, a number of clues from animal models suggest at least some broad categories of genetic etiologies for VACTERL association in humans. First, as animals with mutations in Sonic hedgehog pathway genes (such as *Shh *and the *Gli *genes) have features of VACTERL association, this pathway has long been implicated [38,39]. Teratogen-based animal models further support this hypothesis [40,41]. As humans with loss-of-function mutations affecting the *SHH *pathway have holoprosencephaly, it is unsurprising that patients with isolated VACTERL association do not have *SHH *mutations [42-44]. However, it is certainly possible that perturbations of SHH signaling due to interacting pathways may result in VACTERL association without brain anomalies. For example, mutations/deletions affecting *FOXF1 *(which is linked to SHH signaling) results in a VACTERL-like phenotype (though one small study did not show similar mutations in patients with classic VACTERL association), and a patient has been described with features of VACTERL association due to a mutation in *HOXD13*, a downstream target of *SHH *[45-47].

As VACTERL association appears to be casually heterogeneous, it is unsurprising that several key signaling pathways have also been implicated through animal models. In addition to classic Sonic hedgehog signaling, these include disruption of pathways involving Hox and retinoic acid signaling [48-51]. As many of these pathways interact in complex and still incompletely understood ways, it may be better to think of these pathways as vast intersecting networks involved in developmental signaling, rather than discrete, linear pathways. In other words, a mutation in a gene known to be a key part of one signaling pathway may in fact have multiple effects across a broad network involving many pathways. The fact that patients with Fanconi anemia may be observed to have findings of VACTERL association may provide another clue to more general etiologies, as Fanconi anemia results in congenital malformations, as well as other medical issues such as hematologic disturbances and malignancy, secondary to the accumulation of DNA damage related to chromosomal instability. In Fanconi anemia, the exact nature of the anomalies in a particular patient, including the unilateral nature of some malformations, is thought to relate to stochastic events [52].

As well as classical genetic causes, a number of environmental influences have been implicated, but there is little firm data that would be helpful in counseling patients beyond what is known about teratogens more generally. Reported influences include maternal diabetes, which may result in features of VACTERL association due to multiple factors (these may ultimately be shown to be related to a common pathway). These factors include direct effects of hyperglycemia, oxidative stress and reactive oxygen species, interactions with certain key developmental pathways in genetically vulnerable patients, and, intriguingly, because of patients with genetically-related mitochondrial dysfunction and VACTERL association, mitochondrial damage [53-56]. In addition to maternal diabetes, other reported environmental factors include infertility treatment, and in utero exposure to estrogen and/or progesterone-containing compounds, statins, lead, and, for at least ARM, a number of additional maternal and paternal risk factors and exposures [6,10,11,37,57-59]. Again, these reports must be regarded with caution, as the association between the exposure and the presence of VACTERL association may be speculative. While there is stronger evidence for the association between maternal diabetes and birth defects, the increased relative risk here, as elsewhere, points to a multifactorial etiology in which environmental triggers interact with a genetic susceptibility [60,61].

## Diagnosis and diagnostic methods

Diagnosis of VACTERL association is made on clinical grounds, based on the presence of the congenital malformations outlined above (in the Clinical description section). As described in the section on Definition and diagnostic criteria, the requirements for diagnosis vary among clinicians and researchers. Many (but not all) require at least three component features for diagnosis, without clinical or laboratory-based evidence of the many overlapping conditions, while others emphasize the presence of certain component features, especially TEF or ARM. Another diagnostic approach involves requiring the presence of spatially disparate anomalies (such as occurring both above and below the diaphragm in the same patient) (see Table 1 regarding differential diagnosis) [7-15,19].

As the condition is clinically based, testing techniques recommended for patients suspected to have VACTERL association are outlined in Table 3[22].

**Table 3:** Diagnostic methods.

Feature	Intitial test(s)	Notes
Vertebral anomalies	X-ray; ultrasound and/or MRI of the spine	X-ray may not show subtle spinal anomalies, and will be unable to detect associated anomalies such as tethered cord or syrinx

Anal atresia	Physical examination/observation, abdominal ultrasound for genitourinary anomalies	Additional testing is typically required to define anatomy, especially if concomitant genitourinary anomalies are present

Cardiac malformations	Echocardiogram	Other, more precise techniques, such as cardiac CT or MRI may be helpful to further detail anomalies

Tracheo-esophageal fistula	Physical examination/observation (contrast studies are rarely required)	Patients with VACTERL association but without true TEF may still present with swallowing/breathing anomalies, and clinicians should have a low index of suspicion for confirmatory radiological testing

Renal anomalies	Renal ultrasound	Further testing, such as a voiding cystouerethrogram, may be required in the presence of renal anomalies or if there is other evidence of issues such as vesicoureteral reflux

Limb anomalies	Physical examination, X-rays	Important not to overlook, as the presence of limb anomalies often prompts testing for Fanconi anemia

Suggested testing for patients (in addition to a careful physical examination by an experienced clinician) suspected to have VACTERL association. Specific modalities used should be dictated by the risk-benefit ratio for the specific situation.

CT: computed tomography; MRI: magnetic resonance imaging; TEF: tracheo-esophageal fistula

## Differential diagnosis

The differential diagnosis of VACTERL association is broad, and includes a number of conditions for which genetic testing is available (Table 1). Often, subtle clues on a careful physical examination and family history can help narrow down which conditions are most likely in a patient with features of VACTERL association. For example, autosomal dominant inheritance of certain features may suggest Townes-Brocks syndrome, and the presence of other features not typically seen in VACTERL association may suggest other disorders, such as pigmentary abnormalities in Fanconi anemia, or hypocalcemia in deletion 22q11.2 syndrome. Naturally, this examination is best conducted by individuals who are familiar with VACTERL association and similar disorders.

Ruling-out these conditions is a challenging but critical part of the diagnostic work-up, and is essential for proper genetic counseling, as there is genetic testing available for a number of these overlapping disorders. Ruling-out these disorders will include testing to look carefully for certain features that are not typical of VACTERL association, such as brain malformations, ophthalmologic anomalies, and hearing deficits. As mentioned, some of these similar disorders occur in an inherited manner, though with incomplete penetrance and highly variable expressivity.

Although a controversial issue, it is worthwhile to make special note of Fanconi anemia in the differential diagnosis. There is efficient and relatively affordable testing available for this condition, and it would be important to rule out Fanconi anemia, both for reasons of genetic counseling (as inheritance may be autosomal recessive or X-linked), and also because of the association of hematologic anomalies and malignancies with Fanconi anemia. Proper surveillance for these complications in patients with identified Fanconi anemia could potentially improve outcomes [62,63].

## Genetic counseling

Given the lack of informative data available, genetic counseling for patients and families affected by VACTERL association can be difficult. Approximately 90% of cases of VACTERL association appear to be sporadic, with little increased risk of having multiple affected individuals within a family [36]. However, single or multiple malformations associated with VACTERL association are observed in up to 10% of first-degree relatives of patients with VACTERL association; in other words, there is evidence for an inherited component in a subset of patients [35-37]. Therefore, a careful family history, with further clinical investigation as necessary is a key part of any genetic evaluation for patients with VACTERL association. In families in which multiple members are affected, it is important to note that it is uncommon for all affected individuals to meet full criteria of for VACTERL association. A more common scenario is a proband with VACTERL association who has relatives who may have single component features of VACTERL association, such as isolated (and generally milder) vertebral, cardiac, or renal malformations. The presence of these families points to a complex inheritance pattern involving multiple interacting genetic and environmental factors. Causality in these situations can be difficult to ascertain, which makes accurate genetic counseling challenging. As our understanding of the causes of many congenital malformations continues to grow, patients and families might be counseled to continue to inquire about newly discovered causes and testing modalities that could shed light on their particular situation.

Along these lines, a common and central question that arises during genetic counseling has to do with recurrence risk in affected families. While an exact number is difficult to pinpoint generally, the recurrence risk is likely relatively low as long as similar conditions with inherited forms are ruled-out (such as Fanconi anemia, Feingold syndrome, Holt-Oram syndrome, Townes-Brocks syndrome, and VACTERL with hydrocephalus) [64-69]. Adjustment of the recurrence risk in a given family may be made based on family history, as specific families may appear to segregate VACTERL association type malformations according to a certain inheritance pattern [36].

As with other relatively rare conditions, patient and family-based resources and support groups can be invaluable. For VACTERL association, a number of these groups exist, including: The Pull-Thru Network (United States-based; primarily for patients whose medical issues include ARM: http://www.pullthrunetwork.org); The VACTERL Network (United States-based: http://www.vacterlnetwork.org); TOFS (United-Kingdom based; primarily for patients whose medical issues include tracheo-oesophageal fistula: http://www.tofs.org.uk); VACTERL Association Support Group (United Kingdom-based: http://www.vacterl-association.org.uk).

## Antenatal diagnosis

As with many other conditions, the ability to detect features of VACTERL association prenatally, whether through ultrasound or more sophisticated methods such as prenatal echocardiogram or MRI, is very much dependent on the skill and experience of the medical interpreter. Certain features of VACTERL association, such as ARM or TEF are often not detected prior to delivery, even with frequent and careful prenatal imaging; naturally, this can be distressing to affected families, especially as such malformations can be associated with significant morbidity and mortality.

However, certain clues can suggest VACTERL-type anomalies, such as polyhydramnios and lack of a gastric bubble due to TEF, and a dilated colon due to imperforate anus [34,70]. Other features, such as some types of vertebral anomalies, cardiac malformations, renal anomalies, and limb abnormalities, may be ascertained more easily by antenatal ultrasound [71,72].

It is important to emphasize that the discovery of a single umbilical artery (SUA) may be the first clue to diagnosis. The presence of SUA should always result in a careful antenatal examination for features of VACTERL association as well as for other congenital anomalies [34,73].

## Management

The management of patients with VACTERL association can be complex, and the nuances of treating issues related to each component feature are not covered in this manuscript; the reader interested in a more in-depth discussion might examine some of the referenced articles covering these topics in greater detail. Overall, the management of affected individuals might be thought of as divided into two stages. First of all, conditions that would be incompatible with life, such as severe cardiac malformations, imperforate anus, and TEF, are typically managed with surgery in the immediate neonatal period or as soon as circumstances allow. For example, imperforate anus may be treated with an immediate colostomy, followed later by re-anastamosis and "pull-through" surgery; accompanying genitourinary anomalies are also frequently treated in a staged manner [74]. The correction of cardiac malformations may also require multiple surgeries, depending on the specific type of congenital defect. TEF are typically repaired in a single surgery, though later complications (such as fistula re-occurrence) may necessitate more procedures [25].

Second, many of the congenital malformations can result in longer-term sequelae, as outlined in Table 4. One of the most important themes in managing patients with VACTERL association is recognizing that some congenital malformations may be subtle yet medically important, such as vertebral anomalies that can result in severe back pain later in life [22], or renal anomalies that can predispose to infections, nephrolithiasis, and declining renal function. Managing clinicians must keep these long-term issues in mind [30]. Typically, the role of the clinical geneticist may not involve system-specific outcomes or sequelae after the initial diagnostic period, though some geneticists may find a role in coordination of care.

**Table 4:** Sequelae of malformations associated with VACTERL association.

Feature	Early potential medical complications	Later (post-infant) potential medical complications	Reference(s)
Vertebral anomalies	Scoliosis, tethered cord, syrinx	Progressive scoliosis, back pain, osteoarthritis, tethered cord, syrinx	[21,22]

Anal atresia	Obstruction	Incontinence, constipation, other dysmotility, sexual dysfunction	[22,73,75]

Cardiac malformations	Compromised cardiopulmonary function, dysrhythmias	Compromised cardiac function, dysrhythmias	[19,22]

Tracheo-esophageal fistula	Inability to feed, respiratory compromise, pneumonia	Gastro-esophageal reflux, increased risk of gastro-esophageal cancers (related to reflux), reactive airway disease (can clinically appear similar to asthma, though pulmonary function testing reveals a non-asthma pattern)	[22,74]

Renal anomalies	Vesicoureteral reflux, hydronephrosis, urinary tract infections (also related to anorectal malformations)	Urinary tract infections (also related to anorectal malformations), nephrolithiasis, impaired renal function	[22,30]

Limb abnormalities	Functional impairment	Functional impairment	[21,22]

Logically, the degree of medical complication arising from a particular malformation is highly dependent on the type and severity of the particular malformation.

## Prognosis

With improvements in surgical techniques and in specialized neonatal and post-surgical facilities, the diagnosis of VACTERL association yields a much better prognosis than previously [19,22,74-76]. Nonetheless, even with optimal surgical corrections of malformations such as cardiac anomalies, TEF, and limb abnormalities, patients can face considerable medical challenges throughout life [22,74,76]. Component-specific sequelae are described in Table 4.

Special consideration should be made for ARM. In terms of functional outcome, there is wide variability. However, referral to a highly experienced center with a coordinated, multidisciplinary team, can greatly improve outcomes even among those who have been initially been given relatively grim prognoses regarding issues such as continence. Patients and families, as well as treating clinicians, should be encouraged to seek care through such specialty clinics in order to attempt to achieve the best possible outcome [76].

Finally, despite significant morbidity associated with the component congenital malformations, it is also important to note that patients with VACTERL association do not typically display neurocognitive impairment (in fact, the presence of neurocognitive impairment should strongly suggest an alternate diagnosis) [19,22].

## Unresolved questions

While much remains unresolved, a central and critical question regarding VACTERL association hinges on the causes. Unlike many other conditions whose genetic causes were discovered over the last several decades, the etiologies of VACTERL association remain largely unknown. There are many reasons for this, including likely clinical and causal heterogeneity, the typical sporadic nature of the disease, and the many overlapping conditions. Coupling insights from biological models with newly available genomic technologies may begin to offer more answers about causation in the near future. These answers may then, with more dedicated research, be turned to an even more crucial question: how to improve the health of affected patients and families.

## Declaration of Competing interests

The authors declare that they have no competing interests.
